# Case report: Giant unilocular prostate cystadenoma: A rarer condition with a single cystic mass

**DOI:** 10.3389/fonc.2022.911442

**Published:** 2022-08-12

**Authors:** Wenda Wang, Yu Xiao, Shiyuan Fang, Yi Qiao, Shi Rong, Fengdan Wang, Hao Sun, Zhengyu Jin

**Affiliations:** ^1^ Department of Urology, Peking Union Medical College Hospital, Chinese Academy of Medical Sciences and Peking Union Medical College, Beijing, China; ^2^ Department of Pathology, Peking Union Medical College Hospital, Chinese Academy of Medical Sciences and Peking Union Medical College, Beijing, China; ^3^ School of Clinical Medicine, Chinese Academy of Medical Sciences and Peking Union Medical College, Beijing, China; ^4^ Department of Radiology, Peking Union Medical College Hospital, Chinese Academy of Medical Sciences and Peking Union Medical College, Beijing, China

**Keywords:** prostatic cystadenoma, unilocular, diagnosis, treatment, surgery

## Abstract

Prostate cystadenoma is a rare benign prostatic neoplasm, which grows outside prostate and locates midline between the urinary bladder and rectum. It usually presents as multilocular cysts, thus, named giant multilocular prostate cystadenoma. The definite diagnosis is difficult to be made before surgery, and it depends on histopathology. Here, we report a rarer condition of prostate cystadenoma, which manifests as a giant unilocular cyst with a solid nodule inside. The 55-year-old Chinese male patient presented with dysuria and constipation. MRI revealed a 10.5 × 8.2 cm mono-cystic lesion displacing the rectum to the posterior, prostate, and bladder to the anterior, with a 2.8 × 2.1 cm solid nodule at the anterior wall. ^18^F-FDG PET/CT demonstrated an elevated SUV_max_ (3.5) of the solid nodule. Laparoscopic pelvic mass resection was performed and prostate cystadenoma was diagnosed. In conclusion, when a mass of single locular cyst sits in the male pelvis, the diagnosis of prostate cystadenoma could not be excluded.

## Introduction

Prostate cystadenoma is an extremely rare benign tumor deriving from the prostate. It usually locates between the urinary bladder and rectum and presents as a large multilocular cystic mass, thus, named giant multilocular prostate cystadenoma. The patient usually complain of discomfort, such as lower urinary tract symptoms and defecation problems, and histopathological examination is needed for final diagnosis. Since 1991, fewer than 40 cases of giant multilocular prostate cystadenoma are reported (1, 2). As far as we know, prostate cystadenoma presenting as a unilocular cyst has not been reported in English literature. Here, we report a rarer condition of prostate cystadenoma manifesting as a giant unilocular cyst with a solid nodule, which is challenging for both diagnosis and surgery.

## Case presentation

**Figure 1:**
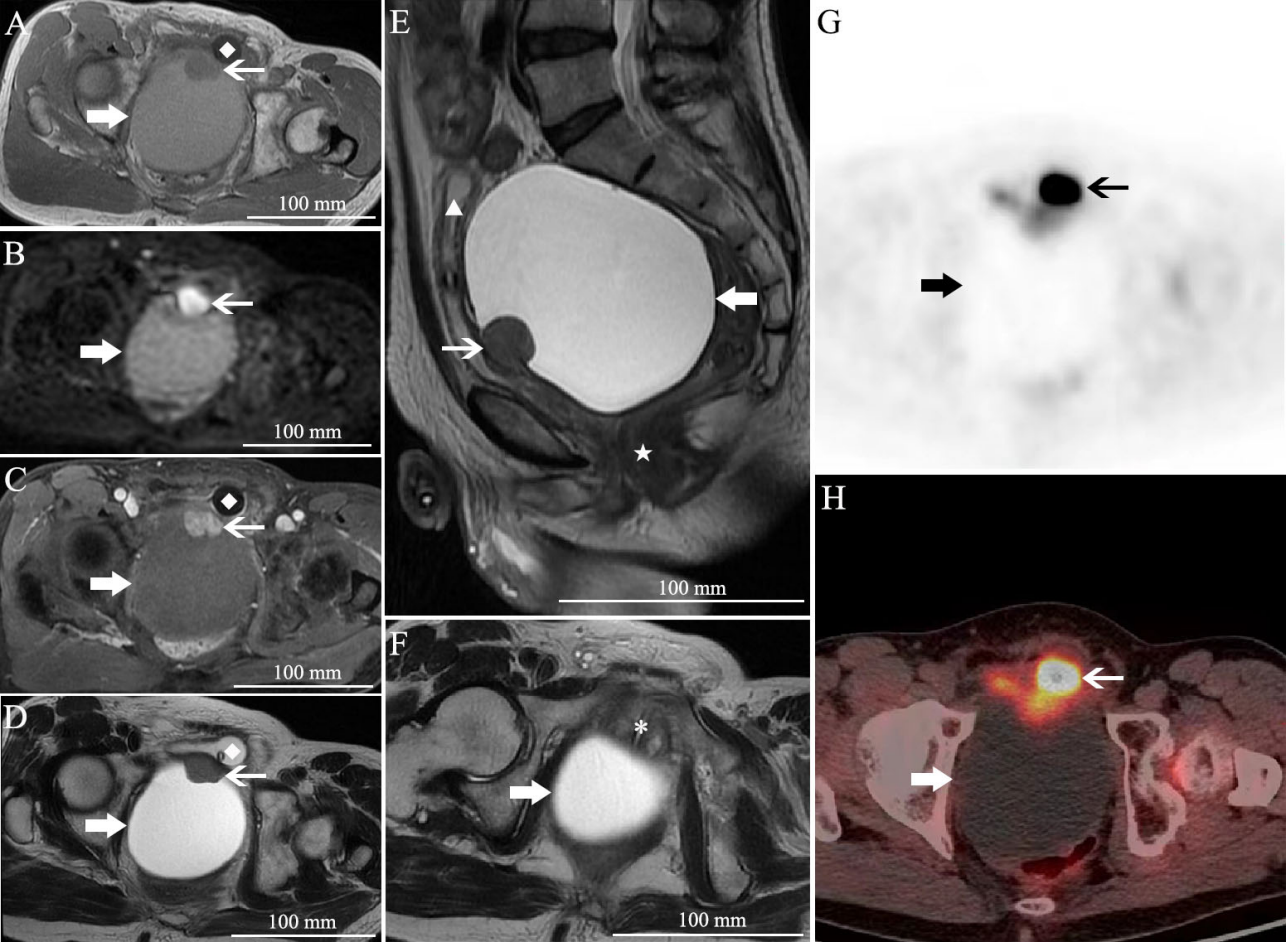
Pelvic MRI and PET/CT. **(A)** Axial T1 weighted image (WI), **(B)** axial diffusion weighted image (DWI), **(C)** axial gadolinium-enhanced fat-saturated T1WI, **(D)** axial T2WI, **(E)** sagittal T2WI, and **(F)** axial T2WI. **(G, H)** ^18^F-FDG PET/CT. There is a giant unilocular cystic lesion measured 10.5 × 8.2 cm in size displacing the rectum (★) to the posterior, prostate (*), and bladder (▲) to the anterior. It was hyperintense on T1WI and T2WI, without diffusion restriction nor enhancement. At the anterior wall, a 2.8 × 2.1 cm solid nodule with isointensity on both T1WI and T2WI, diffusion restriction and marked enhancement, was also noted. PET/CT demonstrated an elevated SUVmax (3.5) of the solid nodule. Please note the catheter in the bladder (◆). The thick arrow, the unilocular cystic lesion; the thin arrow, the solid nodule.

A 55-year-old Chinese male patient presented with dysuria and constipation for 4 months. Four months ago, he experienced severe dysuria and intermitted lower abdominal pain, without fever or hematuria. In the meantime, the patient also developed constipation. Two months later, he experienced constant hematuria and painful urination. Then, he suffered from urinary retention and a urinary catheter was placed to alleviate his lower abdominal pain and hematuria. Physical examination was unremarkable. Urinary occult blood was positive in urinary analysis confirming hematuria. Serum tumor markers including carcinoembryonic antigen (CEA), cancer antigen (CA) 19-9, CA125, CA72-4, and CA242 were all normal. Serum prostate-specific antigen (PSA) was also normal. Pelvic magnetic resonance imaging (MRI) revealed a 10.5 × 8.2 cm mono-cystic lesion displacing the rectum to the posterior, prostate, and bladder to the anterior, with a 2.8 × 2.1 cm solid nodule at the anterior wall (**Figure 1**, Supplement). The cystic component was hyperintense on T1 weighted image (WI) and T2WI, without diffusion restriction on diffusion weighted image (DWI) nor enhancement on gadolinium-enhanced fat-saturated T1WI, indicating protein rich or hemorrhagic fluid. By contrast, the solid nodule, which was isointense on both T1WI and T2WI, showed diffusion restriction and marked enhancement. In addition, ^18^F-FDG positron emission tomography/computed tomography (PET/CT) demonstrated an elevated SUV_max_ (3.5) of the solid nodule. Cystoscope could not be entered due to obstruction caused by the mass.

**Figure 2:**
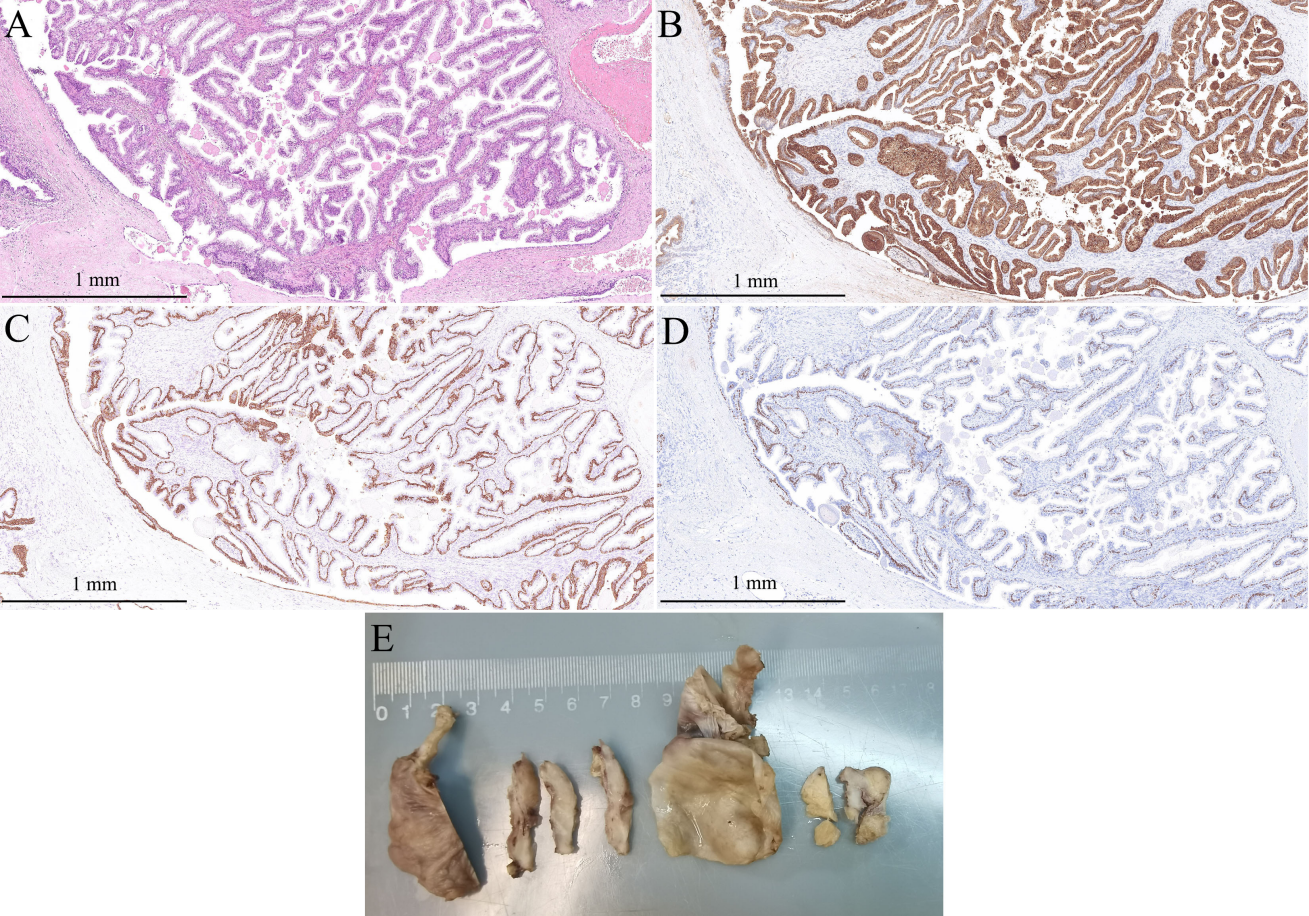
Pathological diagnosis of the mono-cystic mass. **(A)** HE staining slide shows branching papillary structure, lining benign double-layer prostatic epithelial cells (original magnification, ×100); **(B)** The cells lining the cysts were strongly and uniformly positive for PSA (original magnification, ×100); **(C)** Basal cells were immunohistochemical staining for high-molecular weight cytokeratin (34βE12) (original magnification, ×100); **(D)** Basal cells were immunohistochemical staining for p63 (original magnification, ×100); **(E)** The gross specimen.

The diagnosis was difficult before surgery as the lesion was too large and the origin was hard to define. Although there was no obvious invasion of adjacent organs, both MRI and PET-CT suggested that the solid nodule is malignant. However, biopsy under CT guidance was not possible due to the cystic entity and the unavoidable bladder just in front of it. Therefore, laparoscopic pelvic mass resection was performed. During exploration, the giant mass was found to be located between the bladder and rectum, and with marked adhesion to the adjacent organs. Bilateral vas deferens were not able to be detached from the tumor, thus, they were ligated. The cystic capsule was incised from the top, and the brown fluid was aspirated in order to minimize the tumor size. The inner wall of the tumor was smooth, and a solid nodule was noted on the anterior wall. While protecting the rectum from damage, the tumor was completely removed. Pathologically, the tumor is a solitary cystic mass that contains multilevel branching papillary structure, lining benign double-layer prostatic epithelial cells. Overall, the cells lining the cysts were strongly and uniformly positive for PSA and prostate specific membrane antigen (PSMA) and negative for P504S (**Figure 2**, Supplement). Basal cells were identified on light microscopy and with immunohistochemical staining for high-molecular weight cytokeratin CK34βE12 and p63 (**Figure 2**, Supplement). Prostate cystadenoma was diagnosed. The patient was discharged on post-operative day 5, and his symptoms of dysuria, hematuria, and constipation completely resolved 2 weeks after the surgery.

## Discussion

Prostate cystadenoma, a benign prostatic origin tumor, usually presents as a giant multilocular cystic pelvic mass. The age varies from 14 to 80 years old among the patients reported (3, 4). Patients usually complain of symptoms related to mass effect, including lower urinary tract symptoms and defecation problems (5). The level of PSA may be not specific for diagnosis, for PSA value may be elevated or normal (4, 6). On the other hand, PSA level is not associated with tumor size or recurrence, either.

Computed tomography (CT) and MRI usually show a large multicystic mass along the midline between the bladder and rectum. The tumor can be attached to the prostate or entirely separate from the prostate in imaging (7). The septations of the multilocular cyst could be enhanced, and enhanced solid portion may be found in some cases (5). However, our case presented as a large unilocular cystic mass with a solid nodule inside, while no septations were found in the huge cyst. This manifestation of unilocular prostate cystadenoma is rarer. Although the mass located between the bladder and rectum, it was hard to confirm the origin of the mass since it was too large, and the prostate was separated from the lesion and compressed. The normal PSA also increase the difficulty of diagnosis. The diffusion restriction on DWI and elevated SUV_max_ on ^18^F-FDG PET/CT of the solid nodule highly suspected the potential of malignancy. The final diagnosis was made according to the histopathology. The benign PSA-positive epithelial cells on immunohistochemistry analysis confirms the prostatic origin.

Before obtaining the tumor tissue for histological examination, the diagnosis of prostate cystadenoma is difficult. Radiological differential diagnoses of retroperitoneal cystic mass include Müllerian cysts, utricle cysts, and seminal vesicle cysts. It may be easier to distinguish these diseases from typical giant multilocular prostate cystadenomas, for their imaging manifestations are usually not multilocular (2, 8). However, the unilocular cyst of our case is more difficult to be distinguished from them aside from the size. The location of cystic mass between bladder and rectum, and the solid nodule in the cyst may provide considerable and critical information for identification. Other prostatic and retroperitoneal cystic lesions, including cystic change of benign prostatic hyperplasia, prostatic retention cysts, prostatic abscess, and lymphangioma and sarcoma should also be considered for differential diagnosis (5, 9).

Although prostate cystadenoma is a benign tumor, the operation choice is necessary for prognosis. The complete resection is necessary for prevention of recurrence (2, 9). However, the procedure for giant multilocular prostate cystadenomas varies from cystic debulking to pelvic exenteration in previous reports, and the choice depends on the diagnosis and suspicion of benignancy or malignancy and adjacent organ invasion (2). It is reported that gonadotropin-releasing hormone antagonist is effective for recurrence (2, 4, 10).

In conclusion, our case report adds to the recognition that prostate cystadenoma could present as both multilocular and unilocular cystic form. When a mass of single locular cyst sits in the male pelvis, the diagnosis of prostate cystadenoma could not be excluded.

## Data availability statement

The original contributions presented in the study are included in the article/supplementary material. Further inquiries can be directed to the corresponding author.

## Ethics statement

The studies involving human participants were reviewed and approved by the Ethics Committee of Peking Union Medical College Hospital. The patients/participants provided their written informed consent to participate in this study.

## Author contributions

Manuscript writing: WW and YX; Clinical case diagnosis and treatment: WW, YX, FW, YQ, and SR; Data collection and literature resarch: WW, YX, FW, and SF; Manuscript review and revision: HS and ZJ. All authors contributed to the article and approved the submitted version.

## Funding

This study was supported by the Youth Fund of National Natural Science Foundation of China (Grant No. 82001900), and CAMS Innovation Fund for Medical Sciences (2021-I2M-1-051).

## Conflict of interest

The authors declare that the research was conducted in the absence of any commercial or financial relationships that could be construed as a potential conflict of interest.

## Publisher’s note

All claims expressed in this article are solely those of the authors and do not necessarily represent those of their affiliated organizations, or those of the publisher, the editors and the reviewers. Any product that may be evaluated in this article, or claim that may be made by its manufacturer, is not guaranteed or endorsed by the publisher.
